# The association between neighborhood conditions and weight loss among older adults living in a large urban city

**DOI:** 10.1007/s10865-023-00410-0

**Published:** 2023-03-31

**Authors:** Sage J. Kim, Jamine R. Blesoff, Lisa Tussing-Humphrys, Marian L. Fitzgibbon, Caryn E. Peterson

**Affiliations:** 1 School of Public Health, Division of Health Policy & Administration, University of Illinois at Chicago, 1603 W. Taylor St. #781, Chicago, IL 60612 USA; 2 College of Applied Health Sciences, Department of Kinesiology and Nutrition, University of Illinois at Chicago, Chicago, USA; 3 Pediatrics and Health Policy and Administration, Associate Director for Population Science, University of Illinois at Chicago, UI Cancer Center, Chicago, USA; 4 School of Public Health, Division of Epidemiology & Biostatistics, University of Illinois at Chicago, Chicago, USA

**Keywords:** Physical activity, Weight loss, Neighborhood, Crime, Walkability

## Abstract

To elucidate the role of neighborhood walkability and crime on weight loss, we examined data from older adults residing in Chicago who participated in a randomized controlled trial lifestyle intervention. Controlling for individual demographic characteristics and the intervention assignment, the neighborhood homicide rate was significantly associated with weight change. Participants who resided in neighborhoods above the 50th percentile of homicide rate actually gained weight between pre- and post-intervention. On the other hand, there was no significant relationship between the level of walkability and weight loss. Our findings suggest that the social environment related to neighborhood crime may play a more important role in weight loss than the built environment, such as walkability. Urban characteristics related to walkability, such as sidewalks, may increase physical activity, however, interventions aiming to increase physical activity to promote weight loss will benefit by addressing the neighborhood social environment that determines how people navigate space.

## Introduction

The prevalence of adult obesity with Body Mass Index (BMI) greater than 30 is expected to reach nearly 50% of the United States (U.S.) population by 2030 (Centers for Disease Control and Prevention, 2022; Ward et al., 2019). While obesity is associated with multiple deleterious health outcomes, including cardiometabolic diseases and cancers (Afshin et al., 2017; Dixon, 2010; Lauby-Secretan et al., 2016; Wannamethee, Gerald Shaper, Whincup, Lennon, & Sattar, 2011), lifestyle weight-loss interventions have been shown to be beneficial in reducing a variety of these negative health conditions (Anderson et al., 2014; Appel et al., 2011; Elmer et al., 2006; Maruthur, Wang, & Appel, 2009) by improving glucose metabolism and reducing inflammation and oxidative stress, all of which contribute to cardiovascular and metabolic health risks (Esposito et al., 2003; Lofgren et al., 2005; Van Gaal, Wauters, & De Leeuw, 1997).

Studies have documented the effect of neighborhood social conditions, including social support and cohesion, a sense of community, crime and violence, and informal social control, on how people interact with others and with the environment (Bursik & Grasmick, 1993; Sampson, 2003; Shaw & McKay, 1942; Zorbaugh, 1929). Furthermore, neighborhood economic conditions that limit options for healthy good and opportunities for physical activity have been shown to be associated with obesity and weight gain (Black & Macinko, 2008; Coogan et al., 2012; Zhang, Bauer, Powell-Wiley, & Xiao, 2021). As such, neighborhood social and economic conditions determine the built environment (i.e., the physical aspects of where one lives). Research on the built environment has increased significantly over the past decades (Jackson et al., 2013), with an emphasis on how the built environment influences lifestyle behaviors that in turn affect weight management (Brownson et al., 2009; Laddu, Pauluch, & LaMonte, 2021; Scott, Dubowitz, & Cohen, 2009).

The built environment may influence individuals’ health by either facilitating or impeding healthy lifestyle behaviors, including adequate physical activity (Barnett et al., 2017). One important feature of the built environment is walkability. Walkability relates to aspects of physical structures that influence walking as a method of travel U.S. (Environmental Protection Agency, 2021). Studies have documented that people living in more walkable areas tend to be more physically active (Chen et al., 2019; Colley, Christidis, Michaud, Tjepkema, & Ross, 2019) and tend to have lower BMI (Casagrande et al., 2011; Cerin et al., 2018; Creatore et al., 2016; Hoehner, Handy, Yan, Blair, & Berrigan, 2011). Interestingly, however, Zenk and colleagues compared the effects of a weight management program between people living in neighborhoods with variations in the levels of walkability and access to recreational places and found no difference in the program’s effect by the level of neighborhood walkability (Zenk et al., 2019).

Prevalent crime as a component of the built environment, may be a barrier to walkability and physical activity (Gomez et al., 2004; Robinson, Carnes, & Oreskovic, 2016), and yet, research has produced conflicting findings regarding the association between physical activity and neighborhood crime rates (Foster et al., 2014a; Loukaitou-Sideris, 2006; Loukaitou-Sideris & Eck, 2007). While some studies show that higher crime rates and lower reported safety are associated with lower levels of physical activity (Boehmer et al., 2006; Gomez et al., 2004; Richardson, Troxel, Ghosh-Dastidar, et al., 2017; Schoeny, Fogg, Buchholz, Miller, & Wilbur, 2016), other studies have found no effect between these factors and physical activity (Prince et al., 2011; Ruijsbroek, Droomers, Groenewegen, Hardyns, & Stronks, 2015).

Perceptions of neighborhood safety may influence these contradictory findings (Giles-Corti & Donovan, 2002). Perceived neighborhood safety seems to determine levels of physical activity, even after controlling for observed safety, such as neighborhood crime (Janssen, 2014). Other studies have documented the relationship between perceived neighborhood safety, physical activity, and obesity (Bacha et al., 2010; Chaparro, Bilfield, & Theall, 2019; Prins et al., 2013), with higher levels of perceived safety and access to recreational facilities associated with higher physical activity levels among low-income adults (Wilson-Frederick et al., 2014). A meta-analysis found that perceived fear of crime and actual crime rates were inversely correlated with physical inactivity (Rees-Punia et al., 2018). Overall, people who reported feeling safe from crime had 27% greater odds of achieving higher levels of physical activity, whereas those living in areas with high levels of actual crime had 28% lower odds of achieving higher levels of physical activity (Rees-Punia et al., 2018).

Interestingly, Foster and colleagues (Foster et al., 2014a) found that higher levels of certain types of crime, such as burglary and other personal crimes, were associated with increased walkability (Foster et al., 2014a). The authors speculated that walkable conditions may promote walking, while at the same time more people in the public sphere may increase opportunities for crime to take place. There is some evidence to suggest that the relationship between crime and physical activity may vary depending on the type of crime. Although high homicide rates (Kerr et al., 2015) and fear of crime (McGinn et al., 2008) seem to be associated with decreased walking, perceived drug-related crime has been associated with a higher level of walkability (Mason et al., 2013). Overall, the relationship between crime and walkability in current literature seems to be inconclusive.

This study aims to elucidate the role of walkability and neighborhood crime on weight loss using data from older Chicago residents participating in a randomized controlled trial (RCT) lifestyle intervention. This original RCT included predominantly Black older adults who lived on the South Side of Chicago, which is highly racially segregated and affected by increased crime. We hypothesized that neighborhood walkability and homicide would have differential effects on participants’ weight loss and physical activity. Specifically, pre- and post-intervention weight loss would be greater for participants living in neighborhoods with high walkability and lower for participants living in neighborhoods with high homicide rates.

## Methods

### Setting and data

This analysis utilized data from an RCT, *Building Research in Diet and CoGnition* (BRIDGE). Details on the trial have been published previously (Sanchez-Flack et al., 2020; Tussing-Humphreys et al., 2017). Briefly, the BRIDGE trial recruited 185 healthy, predominantly African American older adults over 55 years of age with a BMI greater than 30 kg/m^2^. Participants were randomized into three arms: MedDiet with a weight loss intervention (MedDiet-WL), MedDiet (MedDiet-A) without a weight loss intervention, and a Control group (TDC) with a typical diet without caloric restriction (Tussing-Humphreys et al., 2017). The two intervention groups met once weekly for 8 months, totaling 25 sessions. Baseline data were collected between 2017 and 2019. The study examined the effects of the intervention on cognitive functioning, as well as weight control, dietary intake, and cardiovascular, metabolic, and immune-related biomarkers. The BRIDGE trial was approved by the University of Illinois at Chicago (UIC) Institutional Review Board (IRB# 2016 − 0258) and has been registered at ClinicalTrials.gov (NCT03129048).

### Variables and statistical analysis

Of the 185 original study participants, our analysis included 184 participants who resided within Cook County, Illinois. We geocoded participants’ residential addresses using ArcGIS and appended census tract numbers which were used to merge with the neighborhood variables.

The outcome variable was weight change between pre- and post-intervention (i.e., weight measurement taken eight months after the intervention). Individual-level measures included age (ranging between 55 and 81 years), sex (female vs. male), race (Black vs. all other groups), income (less than $40,000 vs. $40,000 or more), and education (high school or lower, some college, and college or higher). We also included the level of pre-intervention moderate to vigorous physical activity in minutes per day measured by the ActiGraph wGT3X triaxial accelerometer (Santos-Lozano et al., 2012).

Two neighborhood exposure variables were developed: walkability and crime. We used the National Walkability Index developed by the U.S. Environmental Protection Agency, which incorporates three indicators: intersection density (design), proximity to transit stops (distance), and mix of employment and household types (diversity) (Ewing & Cervero, 2010; Hajna et al., 2015; Kärmeniemi, Lankila, Ikäheimo, Koivumaa-Honkanen, & Korpelainen, 2018; Saelens, Sallis, Black, & Chen, 2003; Van Holle et al., 2012). The EPA National Walkability Index ranges between 1 and 20, with scores below 5.76 indicating the least walkable areas, scores above 15.26 indicating the most walkable areas, and a score of 10.50 reflecting average walkability (U.S. Environmental Protection Agency, 2021).

Neighborhood crime was measured using homicide incident cases from the Cook County Medical Examiner’s archive, which includes all homicide deaths along with the address of each incident (Cook County Medical Examiner’s Office, 2021). We geocoded the incident address using ArcGIS and calculated the homicide rates at the census tract level. Walkability and neighborhood crime measures were examined as dichotomous variables, with greater than the 50th percentile treated as high walkability and high crime areas. We appended neighborhood walkability and crime data to participant records, using the participant’s address at the time of enrollment. We conducted descriptive statistics to characterize the sample. To assess differences in weight loss between pre- and post-intervention by walkability and crime, we performed regression analysis. We used Stata version 16 (StataCorp, LLC) for all statistical analyses.

## Results

Table 1 describes characteristics of the study population and summarizes the distribution of participant characteristics by walkability score. The mean age of participants was 66 years, and the majority of participants were female and African American. More than 50% of participants had annual incomes over $40,000 and college or more education. Walkability scores in our sample ranged from 5.7 to 19.0, with a mean of 13.4. Over 91% of study participants lived in neighborhoods with a walkability score greater than the national average of 10.51. Neighborhoods with high walkability scores (above the 50th percentile) had a significantly lower average homicide rate, compared with areas with low walkability scores (below the 50th percentile). Sociodemographic and neighborhood characteristics did not differ by walkability score.

**Table 1:** Descriptive characteristics of participants by walkability

	Total	Walkability	
		High	Low
N	184	96	88
Mean walkability score	13.4	14.9	11.7
Mean crime rate	110.6	95.6	126.9
Mean T1 Weight (kg)^a^	100.5	100.7	100.3
Mean T2 Weight (kg)	96.8	96.7	97.0
T1-T2 diff (kg)	-2.9	-3.1	-2.8
Mean T1 Physical activity	9.5	9.4	9.6
Mean T2 Physical activity	10.0	10.0	10.0
T1-T2 diff	-2.9	0.8	0.2
Mean age	66.3	65.7	66.9
% Female	85.9	84.4	87.5
% Black	90.8	92.7	88.6
% Income >= $40K	52.7	51.6	57.0
% Education > = College	50.5	50.0	51.7
Intervention Group^b^			
MedDiet -WL	40.8	43.8	37.5
MedDiet-A	39.1	39.6	38.6
TDC	20.1	16.7	23.9

^b^MedDiet-WL is MedDiet with a weight loss intervention; MedDiet-A is MedDiet without a weight loss intervention; TDC is the control group with a typical diet without caloric restriction

^a^T1 is pre-intervention; T2 is post-intervention

^e^Moderate to vigorous physical activity was measured by Accelerometer (minutes per day)

**Fig. 1:**
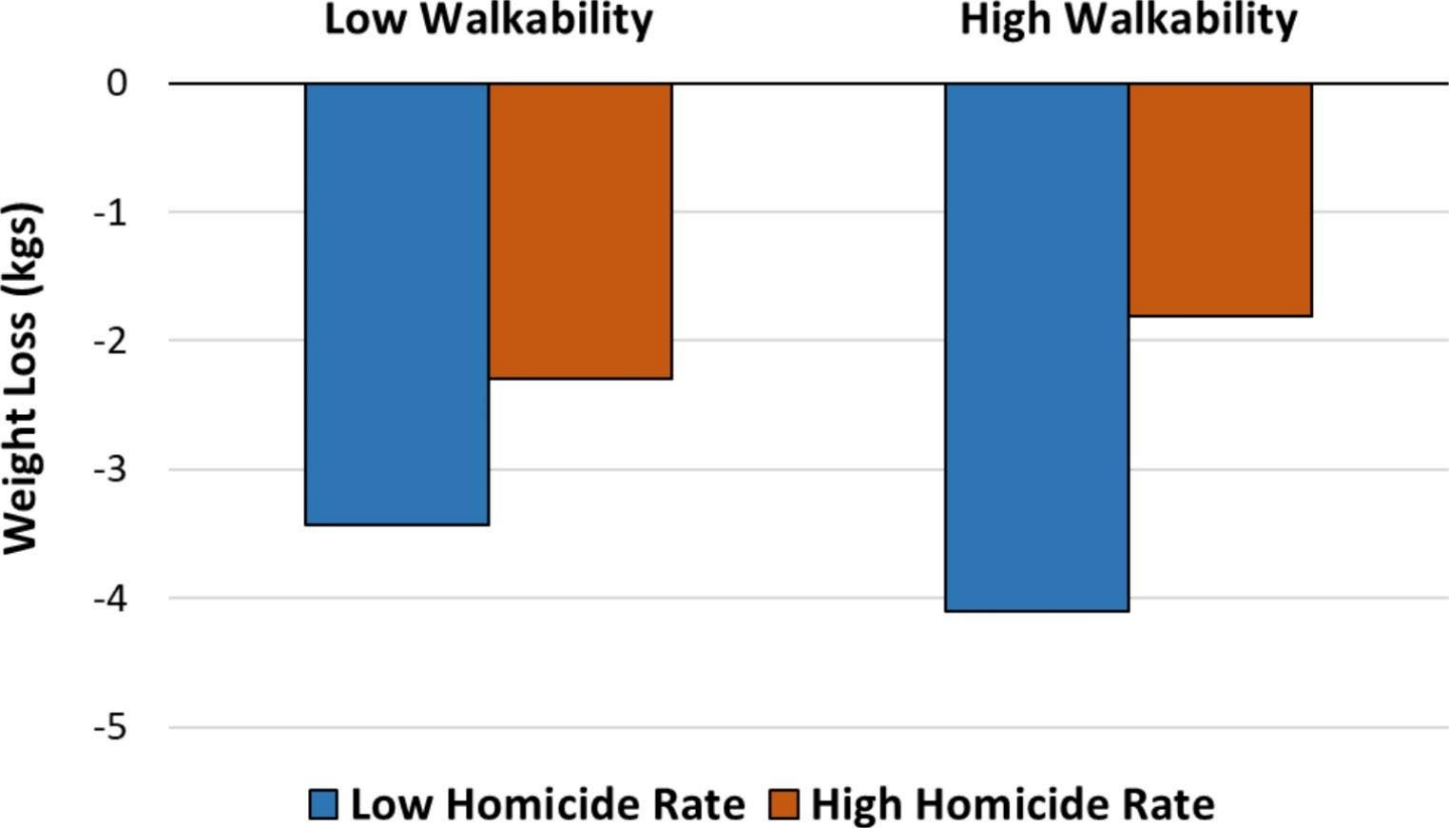
Average weight loss between pre and post-intervention by walkability and homicide rate

Table 2 presents the results of linear regression, with correlation coefficients for walkability score and homicide rate on weight change between pre- and post-intervention. Intervention assignment, age, gender, T1 weight, education, T1 physical activity, and household income were controlled for. Overall, study participants lost an average of 2.93 kg. Walkability score was not significantly associated with a weight change (p < .10). However, the homicide rate was significantly associated with a weight change (p < .05). Participants who resided in neighborhoods with a homicide rate above the 50th percentile lost 1.73 kg less than participants who lived in low homicide areas, controlling for all other confounders. Figure 1 illustrates the marginal effects of walkability and homicide rate on weight loss from the linear regression. Overall participants who lived in low homicide areas lost more weight than those in high homicide areas regardless of walkability. However, among those who lived in low homicide areas, high walkability was associated with greater weight loss than low walkability. For those who lived in high homicide areas, interestingly, high walkability actually was associated with less weight loss than low walkability.

**Table 2:** Linear regression assessing the impact of living in areas with high walkability and high crime on weight loss between pre- and post-intervention

	Coefficient	CI, 95%	p value
Walkability score > 50th	-0.085	-1.491, 1.321	0.905
Homicide rate > 50th	1.731	0.293, 3.169	0.019

Note: Intervention assignment, T1 weight, T1 physical activity, age, gender, race, education, and income were controlled for

### Discussion

Our findings showed that neighborhood crime measured by the homicide rate was significantly associated with less weight loss, controlling for all other variables including the intervention assignment. Participants who resided in neighborhoods at or above the 50th percentile of homicide rate actually gained weight between pre-and post-intervention, regardless of randomization to one of the three intervention arms. On the other hand, the relationship between neighborhood walkability and weight loss was not statistically significant.

Current literature suggests that individuals living in high walkability areas tend to be more physically active. High walkability seems to decrease the risk of excessive weight gain (Lovasi et al., 2009; Rundle et al., 2008). Urban characteristics of walkable areas, such as sidewalks, increase physical activity (Hirsch et al., 2016; Rundle et al., 2016). Thus, policies to improve neighborhood walkability have been promoted for their potential positive impact on residents’ health (Centers for Disease Control & Prevention, 2020). High crime may negate the benefits of a walkable neighborhood environment for physical activity and weight management. Our findings, however, seem to suggest that the social environment, compared with the built environment, may play a more important role in residents’ weight loss. One of the reasons for this might be that the overall walkability score was higher in all Chicago neighborhoods than the national average. Chicago is a large urban city with high density, a mix of real estate types, and well-developed public transportation options (Leinberger & Rodriguez, 2016). In fact, Chicago is consistently ranked among the top-ten most walkable cities in the U.S. (Metromile, 2022; Walk Score, 2023). Indeed, study participants lived in Chicago neighborhoods with higher-than average walkability, and the lack of variability in the walkability score among study participants may have limited our ability to fully examine the contribution of walkability on physical activity and weight loss.

Conversely, high-crime neighborhoods are associated with a variety of poor health outcomes (Sampson, 2003; Won, Lee, Forjuoh, & Ory, 2016), and crime is shown to be a deterrent to residents’ physical activity (Gomez et al., 2004; Mason et al., 2013; Rees-Punia et al., 2018; Richardson, Troxel, Ghosh-Dastidar, et al., 2017; Robinson et al., 2016). Although some studies have found contradicting results (Pratt et al., 2015; Richardson, Troxel, Bhosh-Dastidar, et al., 2017). When aspects of the social environment, such as crime, are considered, the benefits of walkability may be diminished. Neighborhood crime is associated with residents’ fear of crime, which in turn influences engagement in outdoor physical activity. This argument is bolstered by the fact that the majority of neighborhoods within our study had similarly high levels of walkability, but different homicide rates. Among residents living in communities with high rates of homicide, walking may be perceived as dangerous (Jack & McCormack, 2014; Singh, 2016; Van Dyck, Veitch, De Bourdeaudhuij, Thornton, & Ball, 2013). The decision to choose outdoor physical activity may be influenced by homicide rates in the community independent of walkability score.

Although much of the scientific literature finds that urban communities with high walkability may also have high levels of crime (Foster et al., 2014b, 2021; Lee & Contreras, 2021), our findings indicate a more complex relationship between walkability and crime rates. We saw that areas with the highest walkability had lower homicide rates, reflecting current literature. At the same time, to a certain level, higher walkability was correlated with higher homicide rates. Urban communities with high walkability may also have high levels of crime rates (Foster et al., 2014b, 2021; Lee & Contreras, 2021). When walkability is high, residents living in disadvantaged neighborhoods may experience higher levels of violence compared to those in advantaged neighborhoods (Foster et al., 2014b, 2021; Lee & Contreras, 2021). Our findings warrant further investigation into the relationship between social conditions and the built environment, which perhaps interact to influence residents’ health behavior and consequently health outcomes.

## Conclusion

Regular physical activity can positively influence weight management, but not all communities benefit equally from this component of the built environment because walkability is subject to the effects of the social environment, such as high crime. While the built environment represents the physical aspects of where one lives, the social environment creates space for residents to engage with their neighborhood. Interventions aiming to increase physical activity and promote healthy weight management will benefit by incorporating factors of the neighborhood social environment that determine how people navigate space. The social environment related to neighborhood crime may play a more important role in weight loss than aspects of the built environment such as walkability. The social environment and built environment may affect different aspects of urban neighborhood characteristics. Accordingly, urban policy and planning must take into account the interaction between the built and the social environments to facilitate residents’ health and well-being.
