# Correction to: The association between neighborhood conditions and weight loss among older adults living in a large urban city

**DOI:** 10.1007/s10865-023-00430-w

**Published:** 2023-06-13

**Authors:** Sage J. Kim, Jamine R Blesoff, Lisa Tussing-Humphreys, Marian L Fitzgibbon, Caryn E Peterson

**Affiliations:** 1 School of Public Health, Division of Health Policy & Administration, University of Illinois at Chicago, 1603 W. Taylor St. #781, Chicago, IL 60612 USA; 2 College of Applied Health Sciences, Department of Kinesiology and Nutrition, University of Illinois at Chicago, Chicago, USA; 3 Pediatrics and Health Policy and Administration, Associate Director for Population Science, University of Illinois at Chicago, UI Cancer Center, Chicago, USA; 4 School of Public Health, Division of Epidemiology & Biostatistics, University of Illinois at Chicago, Chicago, USA

**Correction to: J Behav Med (2023) 2023 Mar 31**

10.1007/s10865-023-00410-0

The original version of this article unfortunately contained a typo in the third author surname. The author surname was incorrectly listed as Tussing-Humphrys. The correct name should be Tussing-Humphreys.

